# “*Mortui Vivos Docent*” or Who Gives His Body to Science? The Analysis of the Personal Questionnaires of Polish Donors in the Conscious Body Donation Program

**DOI:** 10.1371/journal.pone.0121061

**Published:** 2015-03-19

**Authors:** Grzegorz Bajor, Wirginia Likus, Piotr Kuszewski, Karol Kostro, Andrzej Łoś, Piotr Kłakus

**Affiliations:** Department of Human Anatomy, School of Medicine in Katowice, Medical University of Silesia, Katowice, Poland; ISMETT-UPMC Italy/ University of Catania, ITALY

## Abstract

The Conscious Body Donation Program conducted since 2003 by the Department of Human Anatomy, Medical University of Silesia in Katowice was the first innovative project aimed at obtaining informed donors' bodies for the purpose of teaching anatomy in Poland. The aim of this prospective study was to determine the declared donors' characteristics and to establish the possible motivation for body donation. A total of 244 application files were reviewed and the following information was analyzed: donor’s age, age at which the decision to donate the body was made, donor’s place of residence and declared nationality, family background, education and profession, family structure and religion. Our results showed that mainly elderly people decided to donate their bodies (68.5 ± 11.84 years), living mostly in large and medium-sized cities. Men - donors often lived in small towns. Most of the donors were of blue-collar parentage, completed secondary education and at the time of taking decision to donate where married and retired. Widows were more likely to make the decision to donate than widowers. Most of our donors were Catholic. Our analysis of the profile of Polish donors may be useful to understand better for which groups of people death is not to be perceived as the end, and may become a value, which can be beneficial to living people.

## Introduction

Anatomy, the study of human body composition is one of the first, basic and most important subjects studied by students of medical universities and medical faculties. It forms the backbone for all surgical as well as clinical disciplines. The human cadaver is a crucial tool in the teaching of medical students worldwide [1]. It has been used by anatomists for centuries and attempts to substitute the human cadaver have proven to have a negative effect on the quality of teaching and research in anatomy [2–4]. Not only does cadaveric dissection give a better, in-depth insight into the 3-dimensional structure and topography of the human body but it also helps develop other skills crucial in the future work of medical trainees, i.e. teamwork, respect for human body and proper examination techniques. It cannot be achieved by using anatomical models and computerized learning alone.

In the past years medical schools used numerous sources of human cadavers: unclaimed bodies from morgues, bodies robbed from graves, deceased patients from mental hospitals or penitentiary facilities. Most of such sources are currently illegal [5]. Nowadays, the preferred and most effective method of obtaining cadavers in the Western world is from voluntary body donation programs [6,7], some of which have proven spectacularly successful [8,9].

The voluntary body donation program was started at our center—Department of Human Anatomy, Medical University of Silesia, Poland—in 2003. This form of body donation is a part of, or even a culmination of the first Informed Corpse Donation Program to commemorate Innocent Maria Bochenski. Father Bochenski was a monk, professor, and thinker, his last will was that his body should be donated to science. His body was given to the University of Freiburg, of which Father Bochenski had been a professor for many years. Cadavers are used for teaching students and when they need to be replaced, they are the subjects of special burial ceremony. Poland's first burial of bodies of people who for many years served science and educated the next generation of new physicians was conducted by our University in 2004. Because of the fact that donators had declared various religions the ceremony is accompanied usually by at least two priests: Catholic and Protestant. The university and faculty authorities take part in the ceremony. Usually local TV and press are invited to the ceremony. After the first part of the ceremony held at the Medical University of Silesia, the ashes of the cremated cadavers and remains are placed in The Park of Remembrance at one of local cemeteries. The students of first year of all faculties of the Medical University of Silesia are warmly invited to take part in this ceremony.

We have, so far, obtained over 1250 donors’ declarations, with the numbers rising every year, what makes us proud to run one of the biggest voluntary donation programs in Poland. We have, however, known very little about the profile of our donors.

Therefore we have decided to perform a prospective study to determine our declared donors' characteristics and to establish the possible motivation for body donation in Poland. Our study is the first one that concerns the profile of donors in Poland.

## Materials and Methods

To join the Informed Corpse Donation Program conducted by the Department of Human Anatomy, Medical University of Silesia in Katowice a future donor is requested to fill a form containing a declaration of will to transfer her/his body after death to the Department of Human Anatomy for research and educational purposes. Along with the declaration an anonymous questionnaire was given to each registering person with the invitation to fill in the form and return it to our center. The questionnaires were prepared by the team from the Department of Human Anatomy.

The questionnaire included questions about:

1/ the donor's age, 2/ age at which the decision to donate the body was made, 3/ donor’s place of residence and declared nationality, 4/ family background, 5/ education and profession, 6/ family structure and 7/ religion.

Some questions were either left unanswered or the donors have chosen more than one answer, therefore there are differences in the total number of answers and questions asked. We have received 244 filled questionnaires. The reason for the difference between the total number of declarations of enrollment to the Informed Donation Program and the number of surveys analyzed by us was that only some donors have agreed to fill in the questionnaire.

The statistical analysis was performed using Student`s t-test and nonparametric χ^2^ test. The statistically significant difference between groups has been assessed at the level of p≤ 0.05.

The research study protocol was accepted by the Bioethical Commission of the Medical University of Silesia for ethical consideration of research involving humans.

## Results

### Basic personal data

According to the analysis of basic personal date of whole body donors 57.8% (n = 141) of the registered Polish donors were female, the remaining 42.2% (n = 103) were male. The mean age of all registered donors was 68.5 ± 11.84 years, with female registered donors being at the same age as a male donors (female: 68.76 ± 11.89 years vs. male: 68.05 ± 11.79 years, p = 0.322). The mean age at the time of decision to donate their body to science was 62.55 ± 12.35, with no statistical differences between the two genders (female 62.53 ± 13.96 years vs. male 62.56 ± 11.48 years, p = 0.493). All of the registered donors declared Polish nationality, and Caucasian race.

### Residence and family background

**Fig 1:**
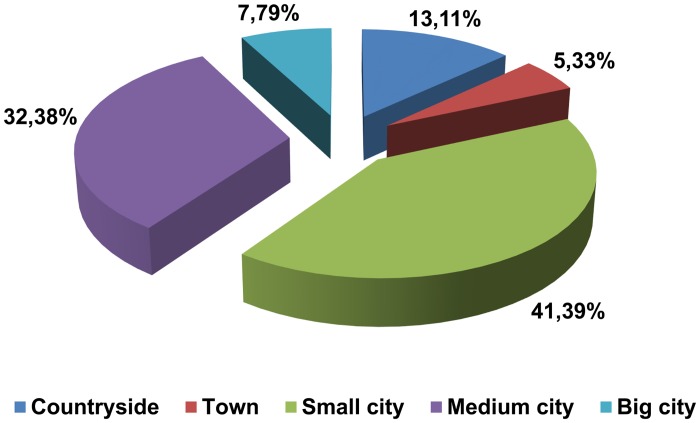
Donors' place of residence (n = 244).

**Fig 2:**
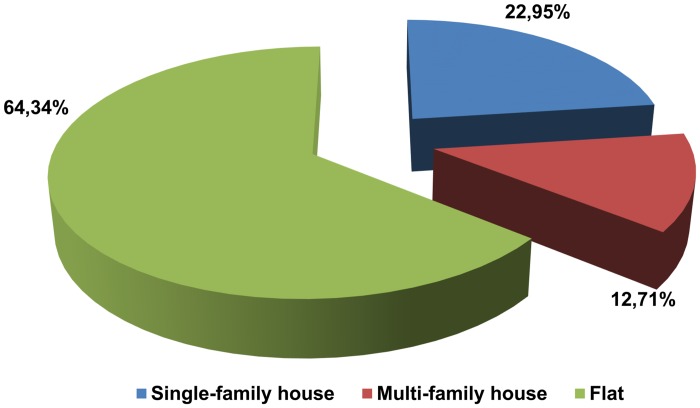
Type of residence (house) among donators (n = 244).

Most of the registrants declared city with <100000 inhabitants as their place of residence (41.39%), the second most common place of residence was a city with >100000 inhabitants (32.38%), followed by countryside (13.11%). The smallest percentage of registrants declared to be living in small towns with <10000 inhabitants (5.33%) and large cities >500000 (7.79%) (Fig. 1). Most of our registered donors live in flats, followed by single-family houses and multi-family houses (Fig. 2). There were no statistically significant differences between the sexes. Comparison of residence between genders proved that among the analyzed group of donators significantly more males (49.51%) than women (35.46%) lived in small towns, p = 0.014. More women declared to be residents of medium-sized cities (36.17%, p = 0.049) and big cities (9.92%, p = 0.016), as compared to men (27.18% and 4.85%).

**Fig 3:**
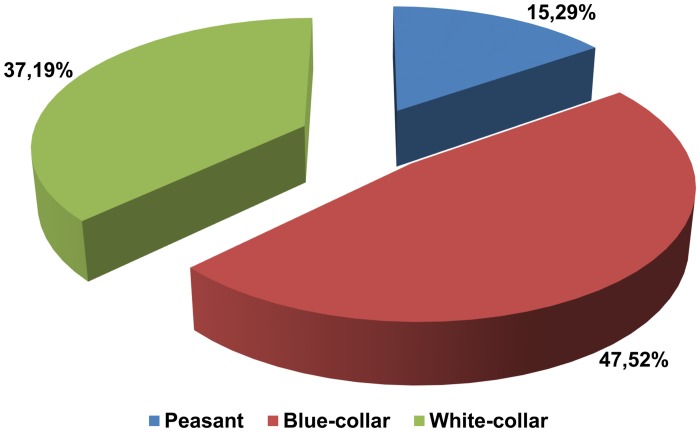
Donors' family background (n = 242).

The analysis of family background revealed that 47.52% of donors declared to be of blue-collar worker descendants, followed by white-collar worker families (37.19%) and peasant families (15.29%) (Fig. 3). Statistically significantly, more men belonged to the blue-collar group (60.20% vs. female 38.30%, p = 0.004). Among those declaring their parentage as white-collars, women predominated (47.52%) in comparison to men (23.30%), p = 0.001.

### Education and occupational activity

**Fig 4:**
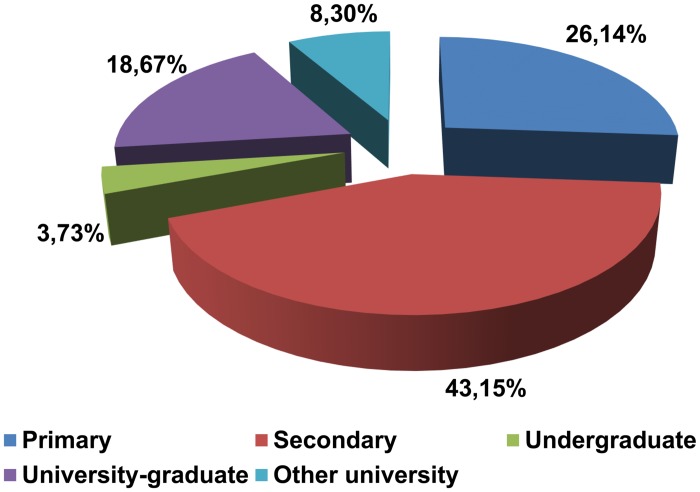
Donors' educational level (n = 241).

The declared education level among Polish donors is shown in Fig. 4. Most of the donors declared completion of secondary education, followed by university graduates. Other university education and undergraduate university education were declared least frequently (8.30% and 3.73%, respectively).

**Fig 5:**
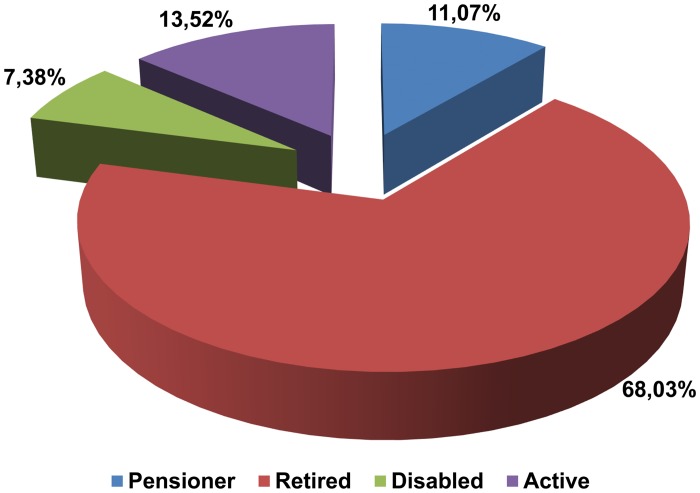
Donors' occupational activity (n = 244).

Analyzing the questionnaires we have shown that significantly more males (36.89%; p = 0.004) acquired a university degree compared with women (17.73%). There were no statistically significant differences between the sexes in other levels of education. Among women, the most common professions were teachers, physicians and economists. Among men, we have not observed such a trend. Only 13.52% of the registrants were still professionally active. The majority of them was retired. Pensioners and disabled formed the two smallest groups (Fig. 5).

Analyzing the differences between sexes, men who statistically significantly more often gave consent to donate their bodies to science were pensioners (17.47%) (p = 0.032) or disabled persons (10.67%, p = 0.041). In case of women the percentage of those groups amounted to 6.38% and 4.96%, respectively. No statistically significant differences have been noted between sexes for other occupational activities.

### Relationship status and family structure

**Fig 6:**
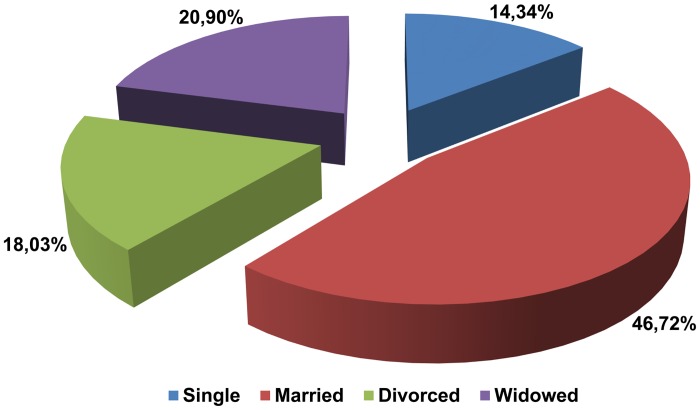
Donors' relationship status (n = 244).

Marriage was the most prevalent relationship status and widowed persons constituted the next most frequent category. Divorced and single were the least often reported marital statuses (Fig. 6). Decisions to donate their bodies for scientific or educational purposes were statistically significantly more often taken by married males than by females with identical marital status (64.07% vs. 34.04%, respectively). Among the widowed population, women took the decision about donation more often (31.91%) than men (5.82%), p<0.001.

**Fig 7:**
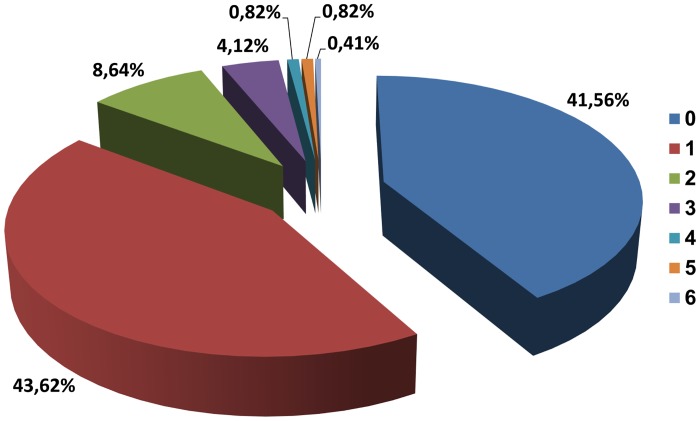
Number of family members living together with the donor (n = 243).

The majority of respondents live either alone or with 1 other family member (Fig. 7). They are statistically significantly more often women (51.77%) than men (27.18%) in case of living alone (p = 0.0001). Others live together with two or less family members (Fig. 7). Among the people living with 1 other family member, 53.4% were men and 36.17% were women. Analyzing donors’ questionnaires, the percentage of men declaring living with 2 other family members was greater than the percentage of women (12.62% vs. 5.67%, p = 0.028), which had statistical significance.

### Religion and faith

A total of 241 individuals answered this section of the survey. Most donors were Catholics (76.35%), followed by atheists (19.50%) and other non-specified beliefs (2.90%). There was only one Orthodox, one Protestant and one Buddhist donor among the respondents. None of the registrants declared Anglicanism, Judaism, Hinduism, or Islam. With 237 donors answering this question, almost the same percentage of donors declared to be practicing (48.52%) and non-practicing (51.48%). Among the people joining the body donation program, women declared to be Catholics (81.14%, p = 0.015) and church-going persons (53.90%, p = 0.006) more often, with statistical significance. As concerns professing a religion in the donor group, no statistically significant differences between the two sexes have been noted.

## Discussion

In the old times, anatomists and surgeons faced problems with obtaining preparations that enabled them to study the human body. Forerunners used various ways to overcome that problem, including use of bodies of deceased criminals, digging bodies out from cemeteries under the cover of the night, or even dissecting bodies of their own family members. Nowadays, neither professors nor students of medicine have such a big problem with studying anatomy on human corpses, as ever more people decide, on voluntary basis, to donate their bodies for scientific purposes. In many countries, including Poland, there are programs of conscious donation of cadavers to be used by departments of anatomy in medical universities. Unlike the situation in the USA, for example, Polish law does not allow private companies to run programs of such kind. Only medical universities are allowed to run them. Informed Corpse Donation Program commemorating Father Bochenski has been run for 11 years now by the Department of Human Anatomy, School of Medicine, Medical University of Silesia, Katowice, Poland. It was the first such innovative project in Poland, with the aim of both informing and obtaining conscious donors for the benefit of studying anatomy. At the same time, the program provides appropriate treatment of the body by students and doctors, including also burial in accordance with the donor’s religion and wish. Our research is the first prospective study in Poland that investigates a number of features of registered body donors. Our analysis covered 244 questioned individuals, it is also one of the largest single-center prospective donor studies in Poland. Not all registrants answered every question. However, taking into account the personal character of some questions, the overall response rate was high. Being a single-center study, this study was limited to the local population.

Most of the donors who decided to donate their body to medicine were in their 60s and 70s, which is similar to numerous studies in other countries [10,11], for example: 70.6 years female, 70.2 years male [12], or 60±15 years for donors in Ireland, 68±13 years for people in New Zealand [13] or 71.4 years in California, USA [14]. The gender profile of the investigated population differs from the population gender structure in Poland, having a higher percentage of registered female donors than the percentage of women in the population. Similar results concerning participation of women in body donation programs were published by Lagwinski *et al*., according to whom women constituted 62% while men 38% of donors [12]. This data stays in contrast with other studies of individuals registering in body donation programs [10,12]. According to Boulware *et al*. men decide to donate their bodies more often than women [15], in contrast to results obtained by other authors [16]. Approximately half of the registrants remained in long-term relationships at the time of decision to donate. Both Cornwall *et al*. and Lagwinski *et al*. [12,13] obtained similar results, with—in case of Cornwall *et al*.—only the Irish population [13] presenting a higher percentage of single donors. Lagwinski *et al*. demonstrated that body donors are mostly single (42%), already retired persons, who never married. Studies by other authors indicate that most frequently the donors were either married or in long-term partnership (41–53%), followed by divorced people (9–32%). 29% of declared body donors from Ireland, 5% from New Zealand and 12% from South Africa were single [13]. Our research demonstrated that body donors are mainly married or divorced persons. A vast majority of individuals who decided to donate their body to our center lived either alone or with only one other family member. This may be related to the age of the donors, when their children have already left home, also many of older donors may be widowed (the second most common relationship status).

A high percentage of Catholic donors in our cohort, together with other beliefs being declared by less than 5% of the individuals and balance between practicing and non-practicing believers reflects the religious structure of the Polish society. However, the number of Catholic donors was 10% lower than in the population, which, coupled with a large number of declared atheists among the registrants, might prove the assumption of impact of religious beliefs and traditional burial ceremonies on the motivation to donate [13,17]. This finds its reflection in the lack of donated bodies in some cultures [7,17–20].

Worth mentioning is also the fact that our center, like many other anatomy schools [13,21,22], organizes and hosts annual commemorating and burial ceremonies. This is a signal to the donors and their families how valuable their gift is. It also might convince some of the more religious donors that their decision does not contradict their beliefs.

We have failed to find any previous studies addressing the issue of place of residence and detailed family background of donors. Therefore we cannot draw any other conclusion than saying that the aforementioned characteristics seem to reflect the general characteristics of the local population [23]. The place of residence, especially small towns and villages, influences social relationships and can impact on taking a decision to donate one’s body. Taking into account the mean age of our donors at the time of completing the survey it is understandable that the vast majority was retired.

The education level of our registrants was generally higher than in the Polish population. Almost three quarters of the donors declared completion of secondary or higher education, with over 30% having completed higher than secondary education. This corresponds with the findings of other authors—Cornwall *et al*. in a multicenter international study noted that the registered donors were generally better educated than the age-matched population. Other investigators have drawn the same conclusions [12,13,24,25]. In the study conducted by Lagwinski *et al*. 63.7% were people who completed secondary education [13], with the exception for the Irish population, there were by far fewer specialists and managers among body donors. Those results differ from the ones obtained by Dluzen *et al*., where 16% of body donors from Northeastern Ohio were professionals regarding occupation [25]. Asad *et al*. evaluated, using discriminative analysis, the donor profile on the basis of questionnaires filled by donors living in the USA. They distinguished two groups: younger educated and married males, predominantly born in the USA, and older divorced or widowed women with university education, whose parents were foreigners [14]. In some cultures, a lower percentage of donors who declare their creed may be related to the traditional perception of burial which should take place soon after death, without cremation of the body [1,26]. Donation of one’s body is connected with deferment of burial, which for many people, for Catholics in particular, may be not in conformity with their religious beliefs. Often incineration of the corpse and the act of body donation are not accepted by the family of the deceased donor. The results of our analyses, concerning donor profile, in comparison with results reported by other authors [27] represent data for a group of Caucasian persons, uniform from the perspective of culture and religion. According to the Polish census data, 97% declare they are Catholics [23]. Attention should be paid to the fact that despite support given by the Catholic church for donating organs for transplantation, whole body donation for scientific purposes is still not commonly accepted. This is mainly due to the cultural conditions and attachment to traditional burial of bodies. However, over the last years, the number of people in Poland who have taken the decision to donate their bodies to science has been increasing (in 2011: 147 acts of body donation, in 2013: as many as 160 acts of donation for our medical university). Some donation programs in other countries may differ in some details. In Korean donation programs a family of the deceased man may choose to oppose the agreed donation. In this program the family can make the decision to donate deceased person’s body [17]. In our program family cannot make the decision of donation. This decision can be made only by a living donor. However if family opposes to the agreed donation, the family’s will is respected.

## Conclusions

Our analysis is the first such analysis of donor profile in Poland. Our results confirm that mainly elderly people, of about 68 years of age, living in big and medium-sized cities prevail in the conscious body donation program. Male donors more often were from small towns. Most of the donors were blue-collars, secondary school graduates. At the time of taking their decision to donate the body, they were already retired, and married. Widows took the decision to donate the body more frequently than widowers.

The analysis of Polish donor profile may be of use in order to understand better who donates the body to science, and for which group of people death does not have to be the end, but can become a value, which living people can make use of. The presented data indicate which social groups seem to be the best target for information campaign. The results of our research may also assist in developing or improving recruiting strategies.

## Supporting Information

S1 Table Survey questions listed by category. (PDF)
