# Supplementary material for: “Mortui Vivos Docent” or Who Gives His Body to Science? The Analysis of the Personal Questionnaires of Polish Donors in the Conscious Body Donation Program
Source: PLoS One. 2015 Mar 19;10(3):e0121061. doi: 10.1371/journal.pone.0121061 (PMC4366327; doi:10.1371/journal.pone.0121061)
Supplement: S1 Table — (PDF) [file pone.0121061.s001.pdf]

S1. Table. Survey questions listed by category.

**Questions about**		**TOTAL (n)**	**Female (n)**	**Male (n)**
**Basic personal data**	Sex, date of birth, nationality	244	141	103
	age of registered donors, decision age	244	141	103
**Donors' place of residence (n=244, female n=141, male n=103)**	*Countryside*	32	20	12
	Town	13	7	6
	Small city	101	50	51
	Medium city	79	51	28
	Big city	19	13	6
**Type of residence (house) among donators (n=244, female n=141, male n=103)**	*Single-family house*	56	29	27
	Multi-family house	31	19	12
	Flat	157	93	64
**Donors' family background (n=242, female n=140, male n=102)**	*Peasant*	37	20	17
	Blue-collar	115	54	61
	White-collar	90	66	24
**Donors` educational level (n=241, female n=139, male n=102)**	*Primary*	63	25	38
	Secondary	104	64	40
	Undergraduate	9	6	3
	University-graduate	45	30	15
	Other university	20	14	6
**Donors` occupational activity (n=244, female n=141, male n=103)**	*Pensioner*	27	9	18
	Retired	166	103	63
	Disabled	18	7	11
	Active	33	22	11
**Donors` relationship status (n=244, female n=141, male n=103)**	*Single*	35	23	12
	Married	114	48	66
	Divorced	44	25	19
	Widowed	51	45	6
**Number of family members living together with the donor (n=243, female n=141, male n=102)**	*0*	101	73	28
	1	106	51	55
	2	21	8	13
	3	10	7	3
	4	2	2	0
	5	2	0	2
	6	1	0	1
**Religion and faith (n=237, female n=141, male n=96)**	*Practicing*	115	76	39
	Non-practicing	122	65	57
**Religion and faith (n=241)**	*Catholics*	184	113	71
	Atheists	47	23	24
	Non-specified beliefs	7	4	3
	Other	3		

n – number of persons choosing each category
